# The GPR17 Receptor—A Promising Goal for Therapy and a Potential Marker of the Neurodegenerative Process in Multiple Sclerosis

**DOI:** 10.3390/ijms21051852

**Published:** 2020-03-08

**Authors:** Angela Dziedzic, Elzbieta Miller, Joanna Saluk-Bijak, Michal Bijak

**Affiliations:** 1 Department of General Biochemistry, Faculty of Biology and Environmental Protection, University of Lodz, Pomorska 141/143, 90-236 Lodz, Poland; angela.dziedzic@unilodz.eu (A.D.); joanna.saluk@biol.uni.lodz.pl (J.S.-B.); 2 Department of Neurological Rehabilitation, Medical University of Lodz, Milionowa 14, 93-113 Lodz, Poland; elzbieta.dorota.miller@umed.lodz.pl; 3 Biohazard Prevention Centre, Faculty of Biology and Environmental Protection, University of Lodz, Pomorska 141/143, 90-236 Lodz, Poland

**Keywords:** GPR17, neurodegeneration, multiple sclerosis

## Abstract

One of the most important goals in the treatment of demyelinating diseases such as multiple sclerosis (MS) is, in addition to immunomodulation, reconstruction of the lost myelin sheath. The modulator of the central nervous system myelination is the metabotropic receptor coupled to the G-protein: GPR17. GPR17 receptors are considered to be sensors of local damage to the myelin sheath, and play a role in the reconstruction and repair of demyelinating plaques caused by ongoing inflammatory processes. GPR17 receptors are present on nerve cells and precursor oligodendrocyte cells. Under physiological conditions, they are responsible for the differentiation and subsequent maturation of oligodendrocytes, while under pathological conditions (during damage to nerve cells), their expression increases to become mediators in the demyelinating processes. Moreover, they are essential not only in both the processes of inducing damage and the death of neurons, but also in the local repair of the damaged myelin sheath. Therefore, GPR17 receptors may be recognized as the potential goal in creating innovative therapies for the treatment of the neurodegenerative process in MS, based on the acceleration of the remyelination processes. This review examines the role of GRP17 in pathomechanisms of MS development.

## 1. Introduction

Neurodegenerative diseases are characterized by the gradual and progressive loss of nerve cells. Multiple sclerosis (MS) is a severe autoimmune disorder that depends on many factors and changes over time [1]. Most people with MS are diagnosed between the ages of 20 and 40, with at least two to three times more women being diagnosed with the disease than men. MS-related disability limits participation in everyday activities and has a considerable impact on quality of life, thereby affecting productivity and employment. Due to the constantly growing number of patients, this disease has major socio-economic consequences and is a significant public health problem [2,3].

The pathological hallmark of MS is multiplicity focal areas of myelin loss within the CNS, called plaques or lesions, which are mainly located within the white matter, usually in the periventricular area and within the brainstem and spinal cord. MS is an autoimmune disease whereby the body’s immune system mistakenly attacks myelin in the central nervous system (CNS) and so is characterized by demyelination of white matter fibers due to the disintegration of the myelin sheath. MS progression is characterized by white matter plaque formation and axonal injury, which mainly occur in the spinal cord, optic nerve, brain stem, and periventricular regions [4]. Despite the fact that MS traditionally is regarded as a white matter disease, it is well demonstrated that demyelinated lesions can occur also in grey matter [5]. There are studies using immunohistochemistry and confocal microscopy that have confirmed the presence of inflammation and neuronal pathology of the cerebral cortex in MS. Furthermore, cortical lesions have exhibited extensive neuronal injury including neuritic distensions and dendritic or axonal transactions. The focal grey matter injury and subsequent tissue atrophy are already present at the early stages of MS and evolve faster than white matter pathology. In addition, grey matter damage has been correlated with physical disability and cognitive dysfunction [6,7]. MS is associated with disruption of the blood-brain barrier (BBB) and invasion of peripheral immune cells (especially autoreactive T cells and macrophages). This induces an inflammatory cascade that results in the formation of new demyelinated plaques and axonal loss [8,9]. Moreover, B cells have been confirmed to be among the numerous factors that affect MS development and severity by acting as a source of antibody-producing plasma cells, functioning as APC controllers as well as producing immune-regulatory molecules [10].

MS can present in four different forms: clinically isolated syndrome (CIS), relapsing-remitting MS (RRMS), primary progressive MS with gradual progression of disability (PPMS), and secondary progressive MS (SPMS). The most commonly diagnosed form in patients is the RRMS type (about 90%). Initially, the disease is characterized by reversible episodes of neurological shortage, followed by gradual and irreversible deterioration overtime [11]. Statistical data indicates that within 20–25 years, around 60–70% of RRMS patients on average transform into SPMS, which is characterized by a progressive and irreversible neurological decline [12,13]. The symptoms of MS are quite variable and depend on the patient, but generally involve sensory disturbance, bladder dysfunction, cognitive deficits, unilateral painless loss of vision, double vision, limb impairment, ataxia, fatigue, and digestive disorders [14]. This leads to considerable disability through deficits of sensation and of motor, autonomic, and neurocognitive functions [15].

There currently exist disease-modifying therapies (DMTs) for patients suffering on MS that can modulate and/or suppress many different mechanisms of autoimmunity, with a strong influence on the course of the disease. Existing available DMTs only have a positive effect on the RRMS form, however, and no beneficial effect on the progressive form has been observed. DMTs approved by the European Medicine Agency and Food and Drug Administration that affect the course of MS include dimethyl furamate (DMF), natalizumab, fingolimod (FTY), interferon beta (IFN-β), glatiramer acetate (GA), and teriflunomied (TFM). IFNs, GA, TFM, and DMF are considered first-line therapies, while specific monoclonal antibodies (mAb) are considered second- or third-line drugs [16,17].

The pathogenesis of MS is characterized by a cascade of pathobiological events ranging from focal macrophage, leukocyte infiltration, and microglia activation, as well as the antigen-dependent activation of B lymphocytes and antibody-generation by plasma cells, to demyelination and chronic axonal degeneration [18,19]. The most likely immunological mechanisms involved in the pathogenesis of MS include molecular mimicry and the bystander activation effect [20]. Molecular mimicry is a phenomenon that involves the activation of B and T cells in response to actions directed against their own antigens by pathogen-like products that are similar in sequence and structure. The bystander activation is the spread of inflammatory processes to neighboring, healthy cells, due to the activation of autoreactive T lymphocytes circulating in the body [21]. MS pathophysiology is accompanied by the strong activation of astrocytes and microglia cells, as well as the massive infiltration of immune cells to the site of the demyelinating lesions. In the MS course, CD8+ T cells outnumber CD4+ T cells in the area of active demyelinating lesions. CD4+ T-cell-mediated autoimmunity has long been accepted as one of the most important aspects of MS pathogenesis, especially in the initiation of the disease [22,23]. Also, T-helper type 1 (Th1) cells, characterized by the production of interferon gamma (IFN-γ), are considered a type of effector Th cell that mediates the pathogenesis of MS [24]. Th17 cells are at least as crucial as Th1 cells in this disease [25,26].

The characteristic feature of MS is the dissemination of plaques expanding the area of primary demyelination. However, MS can be difficult to diagnose because there is no single test to distinguish MS from other demyelinating disorders such as myelinoclastic diffuse sclerosis (Schilder disease), neuromyelitis optica (NMO), or transverse myelitis. The methods of diagnosis mainly based on McDonald’s criteria from 2017 involve evidence from a clinical examination, medical history, lab tests, and magnetic resonance imaging (MRI) of the brain and possibly the spinal cord [27,28]. A key rule for diagnosing MS is to expose evidence that demonstrates chronic, multifocal demyelination of the CNS (brain and spinal cord) with clinical and/or radiological evidence of ‘dissemination in space’ (DIS, demyelination lesions in more than one place in the nervous system) and ‘dissemination in time’ (DIT, demyelination lesions have occurred more than once). Massive infiltrations of immune mononuclear cells through the BBB into the CNS cause the perivascular spreading inflammatory lesions. Aforementioned inflammatory changes observed in the earlier stages of the disease result in the formation of new demyelinating lesions, constituting one of the most important and major pathological hallmarks of MS. Chronic inflammation leads to the disturbed functioning of oligodendrocytes for demyelination, which disrupts the signaling between the neurons [29,30]. In the early stages of the disease, demyelination mainly affects the myelin sheath surrounding the nerve fibers formed by oligodendrocytes, whereas neurons and axons are unaffected by degenerative changes. In the primary stages of MS development, only partial remyelination of the myelin sheaths occurs. Remyelination is one of the best-documented examples of tissue repair in the human adult CNS. The result of this process is the activation, proliferation, and migration of oligodendrocyte precursor cells (OPCs) towards the demyelinated axons, resulting in myelin sheath reconstruction. The myelin sheath, rich in fatty lipids and possessing a lower water content, enables energy-efficient saltatory conduction, and provides essential trophic support in maintaining axonal integrity and survival [31]. The brain shows neuroplastic abilities to compensate for existing functional defects. However, the newly formed myelin is much thinner than the original, and is unable to perform its functions properly. Constantly recurring relapses weaken the remyelination process, leading to permanent damage to the axon and the formation of a scar around it, called a demyelination plaque [32]. Due to the dynamic and heterogeneous development of the disease, there are three types of plaque: active chronic, active acute, and inactive. An active plaque demonstrates marked effects in the development of the inflammatory demyelinating process, and activation of microglia. It is also characterized by the presence of clusters of reactive T lymphocytes, macrophages, plasma cells, cytotoxic cytokines, reactive oxygen species, and activated complement components, which cause damage to the myelin sheath [33,34].

Despite the fact that the process of myelin sheath destruction is well known, the mechanisms for converting healthy white matter into fully demyelinated lesions has not been absolutely elucidated. A complete understanding of the functioning of these mechanisms could perhaps revolutionize the treatment of neurodegenerative diseases, including MS. A promising goal here seems to be the recent intensively studied seven-transmembrane domain receptors (GPR17). What is more, the proven dual role of GPR17 receptors, distinct in time and space in different types of CNS cells, has allowed them to be considered an ideal marker of dynamically variable demyelination and remyelination processes in the development of MS. The development of effective biomarkers that will predict treatment response and inform prognosis based on the degree of disease activity would allow for individualized clinical management of patients with MS. Thus, the optimization of treatment in the initial phase of the disease, validated via specific biomarkers, would greatly inhibit disease progression and the development of disability [35].

The MS biomarkers may be divided into clinically used and potential biomarkers, which additionally are subdivided into inflammatory (cytokines and chemokines), and degenerative, and reflect different pathological processes such as axonal degeneration, astrogliosis, or oxidative stress [36]. The choice of the proposed MS biomarker candidate using a specific ‘biomarker validation’ for characteristic disease entity should be considered carefully. Finding the most reliable potential biomarker of neurodegeneration in MS has been of considerable interest over the last two decades [37]. The detection of a pathological process must be carried out in a sensitive and specific way. The cerebrospinal fluid (CSF) is the most direct origin of biomarkers given its closeness to disease pathology [38]. However, CSF biomarkers of neurodegeneration and neuro-restoration do not permit assumptions to be made about the pathogenesis of MS, they focus only on intrinsic neurobiological processes that are not necessarily unique to MS. They offer the opportunity to monitor pathological processes linked to the course of a disease. Blood-based biomarkers have essentially been used only for differential diagnostic tests and for safety concerns associated with DMT treatment [39]. Nonetheless, with certain exceptions, plenty of suggested biomarkers are strictly interdependent but have yet to be shown to have any prognostic usefulness in MS. Furthermore, numerous markers are not specific to MS. Whereas these markers are interesting in terms of explaining the molecular mechanisms of disease progression, most of them have not translated into practical use. Actually, the identification of predictive biomarkers in the course of MS is relevant and remains crucial in this field. Thus, there is an urgent need to discover the diagnostic marker of disease activity or disability progression, especially in MS, which is still not curable [40].

## 2. General Characteristics of G Protein-Coupled Receptors

G-protein coupled receptors (GPCRs) are a large family of surface receptors involved in intercellular signaling [41]. Sequence analysis allows about 800 GPCRs to be identified in the human genome [42]. GPR receptors constitute about 2% of the human genome and still remain a target for pharmacological research associated with the discovery of new and effective drugs [43]. GPRs comprise a 7-transmembrane α-helix connected by three external- and intracellular loops. The extracellular region, which is responsible for ligand binding, also includes the N-terminal site. The intracellular region interacts with guanine nucleotide-binding proteins (G proteins), and includes a C-terminal site that has kinase activity, which enables them to auto-phosphorylate [44]. GPCR participation in creating a second messenger is significant in creating abundant pathways of cellular signaling. Activation of GPR is strictly associated with a heterotrimeric G protein. This G protein is composed of a Gα subunit and Gβγ subunit dimer. In its inactive state, Gα is bound to a molecule of guanosine diphosphate (GDP). After connecting with the ligand, an activated GPCR catalyzes the conversion of GDP for guanosine triphosphate (GTP) on the α-subunit [45]. The binding of the GTP molecule causes conformational changes in the Gα protein, which results in the functional separation of the Gα and Gβγ subunits and affects the activity of effector proteins. Both Gα-GTP and Gβγ can activate effector proteins and various signaling cascades, while the receptor is able to activate the next G protein [46]. All of the GPCRs couple to three types of Gα-proteins: αs, αi, and αq/11. αs and αi stimulate or inhibit adenylate cyclase (AC) activity, while αq activates phospholipase C (PLC) [47]. The Gα subunit activates the AC-dependent pathway by stimulating or inhibiting the production of cyclic adenosine monophosphate (cAMP), using a high-energy adenosine triphosphate (ATP) molecule, reached by straightforward stimulation of the membrane-associated enzyme AC. Subsequently, cAMP can act as a second messenger that goes on to interact with and activate protein kinase A (PKA) [48]. Furthermore, Gα stimulates the membrane-bound PLC, which then cleaves phosphatidylinositol 4,5-bisphosphate (PIP2) into twosecond messengers: inositol trisphosphate (IP3) and diacylglycerol (DAG). Gα is also involved in the Rho protein family GTPase signaling pathway, which is involved in controlling cell cytoskeleton remodeling, and thus in regulating cell migration [49]. There are at least 20 different Gα subunits, which separate into four major types. The division, role, and potential effects of these Gα subunits are presented in Table 1.

Gβ and Gγ subunits can recruit protein into the plasma membrane, such as G-protein coupled receptor kinases (GRK), and can also regulate ion channel activity to generate further cellular responses. Return to resting state of G proteins occurs by hydrolysis of the phosphate group from the GTP molecule [63]. The primary role of GPCRs in physiology is to facilitate cell communication through recognition of diverse ligands, including bioactive peptides, amines, nucleotides, and lipids. GPCRs also mediate our sense of vision, olfaction, taste, and pain [64]. Currently, there are several classification systems in use by GPCRs. One widely used system is the A–F system, which groups GPCRs into six classes based on sequence homology and functional similarity: rhodopsin-like (Class A); secretin receptor family (Class B); metabotropic glutamate/pheromone (Class C); fungal mating pheromone receptors (Class D); cAMP receptors (Class E), and frizzled/smoothened (Class F) [65]. Another classification is the GRAFS system, which divides the GPCRs into five groups: rhodopsin (R); adhesion (A); glutamate (G); secretin (S), and frizzled/taste2 (F) [66]. In this system, over 90% of the receptors are allocated to the rhodopsin-like family [67]. Based on a phylogenetic analysis, rhodopsin-like family receptors can be divided into 19 subfamilies (A1–A19). Rhodopsin-like receptors affect many autocrine, paracrine, and hormonal processes [68]. The second largest GPR receptors include adhesion receptors. A number of adhesion GPCRs can have important roles within the immune system and in nervous system development. For example, the epidermal growth factor-like molecule containing mucin-like hormone receptor (EMR2) is expressed by myeloid cells (including monocytes and dendritic cells), and has been shown to be involved in the activation and migration of human neutrophils [69]. Glutamate is an important neurotransmitter in many excitatory synapses in the mammalian brain. Consequently, glutamate receptors have been examined in detail for having principal roles in synaptic transmission and development of neurons [70]. The secretin receptors belong to classic hormone receptors, some of which have a neuroprotective effect on the CNS [71]. The last class of GPCRs includes frizzled/taste2 receptors activated by wingless/int (Wnt) proteins, and their function is based on the control of many cellular processes such as neurogenesis, as well as neural crest development. The frizzled/taste2 receptors control cell death, proliferation, and membrane polarity by mediating signals from the secreted glycoproteins Wnt [72]. One exemplary receptor is the taste 2 (TAS2) receptor. The role and function of this receptor is fairly unclear, but it is known to be expressed in the tongue and palate epithelium, and is likely to function as a bitter taste receptor [73].

## 3. The Essential Role of GPR17 in Neurodegeneration

In 1996, Raport et al. were the first to isolate the *GPR17* gene (referred to as R12) while attempting to identify new rhodopsin-like receptors for chemokines signaling through the G protein [74]. In 2001, its first mentions in the literature described GPR17 as an orphan receptor with an unknown ligand [75,76]. GPR17 was characterized for the first time in 2006 [77]. Phylogenetically, GPR17 is closely related to two different receptor families: the purine P2Y subfamily (P2Y1 and P2Y2), and cysteinyl leukotriene (CysLTs) receptors (CysLT1 and CysLT2). GPR17 also shares a 28%–40% amino acid identity with them [78]. The GPR17 receptor is classified into the rhodopsin-like family, together with the purinergic P2Y receptor [79]. GPR17 is a type of receptor precisely activated by both uracil nucleotides (UDP, UDP-glucose, and UDP-galactose) and CysLTs (LTD4 and LTC4) [80]. The endogenous ligands for GPR17 named above are released extracellularly from damaged cells at sites of inflammation, where the GPR17 expression is evidently elevated around the lesions. This indicates that GPR17 plays a large part in brain damage during neuroinflammation conditions [78]. In humans, the gene for GPR17 is located on the chromosome 2q21. The *GPR17* gene contains three exons and two open reading frames (ORFs). As a result of alternative splicing, two isoforms of these proteins arise: a short isoform containing 339 amino acid residues, and a long isoform with 367 amino acid residues [81].

In both physiological and pathological conditions, GPR17 receptors participate in nucleotide signaling and activation of microglia, and are responsible for the regulation of many biological processes occurring in the living cells of the nervous system such as astrocytes and oligodendrocytes [82]. Disorders in nucleotide signaling are associated with many human diseases, including acute ischemia and stroke, brain and spinal cord injuries, and chronic diseases of the nervous system such as MS [83]. Intensive research in recent years has shown that GPR17 receptors are abundant in the nervous system, including the frontal cortex, striatum, brainstem, and medulla [84].

During brain injury, there is a local repair response and a deep remodeling aimed at recreating the most important functions of the nerve tissue. This process is characterized by rapid activation of microglia, production of pro-inflammatory mediators, and infiltration of numerous types of inflammatory cells such as oligodendrocytes, T cells, and macrophages into the damaged brain tissue. The repair process requires cooperation between the damaged neurons releasing the warning signals, and the glial cells responding to these signals [85].

Oligodendrocytes are glial cells that are responsible for myelination of axons in the CNS, which, through supporting the neurons in maintaining their functions and providing metabolic and trophic supply at the axon-myelin unit, are a leading contributor to the prevention of neurodegeneration [57]. Oligodendrocytes and their extensively distributed progenitor cells greatly influence and control processes shown to be commonly dysregulated in neurodegenerative diseases [86]. It is widely known that the presence of oligodendrocytes and an intact myelin sheath are necessary for the proper functioning of neurons [87]. Myelination is an extremely organized process in which numerous factors influence the timing of oligodendrocyte maturation and, as a last resort, the myelination of the axon. This process is closely controlled by a complex intracellular program to differentiate oligodendrocytes as well as neurons [88]. Secretion of growth factors such as glial- and brain-derived neurotrophic factor (GDNF and BDNF) from oligodendrocyte granularity can modulate axonal outgrowth. These examples highlight the importance of oligodendrocytes and myelin in the preservation of the physiological function of neurons. Critical to this process is the switch from a proliferative/migratory state to the exiting cell cycle, and differentiation into non-dividing, non-migratory mature oligodendrocytes [89].

Microglia cells (monocytes) migrate to damaged sites, secrete pro-inflammatory mediators such as chemokines (e.g., cytokine-induced neutrophil chemoattractant (CINC) and monocyte chemoattractant protein 1 (MCP-1)) [90], cytokines (e.g., IL-1 and IL-6), tumor necrosis factor (TNF)-α, transforming growth factor (TGF)-β [91], and arachidonic acid metabolites. They also activate the purinergic signaling system, thus contributing to the repair of damaged nerve cells [92]. During CNS development, the generation of oligodendrocytes and myelination are closely regulated, and the failure of myelination or remyelination—as happens in MS—finally results in loss of axonal function. OPCs proliferate and differentiate into mature oligodendrocyte cells that carry out remyelination processes, restoring re-communication between neurons [93]. OPCs, also known as NG2+ cells due to their expression of the proteoglycan NG2, are well known for their proliferation and participation in remyelination [94]. The NG2 chondroitin sulfate proteoglycan has been proven to be one of the most solid commonly used markers for OPC maturation in the CNS [95]. NG2-glia is a subtype of glial cells in the CNS that encompass approximately 3–4% of cells in the grey matter and 7–8% in white matter, making them the fourth group of glials after astrocytes, microglia, and oligodendrocytes [96,97]. GPR17 is expressed in oligodendrocyte lineage cells, and their level is augmented during the differentiation of OPCs into pre-immature oligodendrocytes [98]. Therefore, they are considered a key timer of oligodendrogliogenesis [99]. Comparisons of postnatal cerebellar myelination in wild-type and NG2- mice reveal diminished numbers of OPCs in the developing white matter of the NG2- mouse, as well as delayed myelination [94]. Mature OPCs that migrate to lesions are effective in generating myelinating oligodendrocytes to repair lesions in the early stages of demyelinating pathologies [100]. The process of remyelination in MS requires the differentiation of OPC cells into pre-oligodendrocytes, and eventually transform into mature myelinating oligodendrocytes capable of renewing the myelin sheath [101]. Many studies have shown that remyelination is possible within MS lesions; however, such extensive remyelination has only been observed in a few cases [102]. Remyelination is an individual process that in some patients is present in many lesions, while in some it is restricted only to the margins of a few lesions. Another factor affecting the course of remyelination is the duration of the disease. The process of remyelination is present in active lesions at an early stage of MS, while during the late course oligodendrocytes cannot keep up with the myelin superstructure due to overly extensive changes, suggesting that the mechanisms regulating remyelination are insufficient to reach complete reconstruction of the myelin sheath [103]. At the late stage of MS, remyelination is probably limited by oligodendrocyte density. Failure of sufficient oligodendrocyte recruitment, especially to lesion cores, is a significant reason for the weak remyelination of lesions. It has been observed that at an early stage of the disease, demyelinating lesions are rich in a large number of OP cells and pre-myelinating oligodendrocytes, but fail to differentiate into mature oligodendrocytes, which suggests that the remyelination process is blocked at the pre-myelinating stage in demyelinating lesions [101]. The process of remyelination begins with the immediate reaction of OPC cells to interruption of the sheath through activation and colonization at the site of damage. Where they eventually differentiate into mature oligodendrocytes capable of building myelin [104]. The pathology of the blockade of OPC differentiation is considered to be the main cause of disorders in the remyelination process that are observable in neurodegenerative disorders [105].

Cerebral ischemia promotes the activation of GPR17+ oligodendrocyte precursors, which begins proliferating in the area of demyelinating lesions. In the early stage of damaged of CNS tissue (within 24 h), an increase in the GPR17 level in damaged neurons and significant growth in the mortality of GPR17+ neurons, as well as oligodendrocytes, produce observable damage [80]. Studies conducted on rodents have shown that blockading of the GPR17 receptors through typical P2Y/CysLT antagonists, and inhibition of *GPR17* gene expression, significantly limits the necrosis of CNS tissue. The results obtained suggest that GPR17 receptors could be molecular markers associated with induced neuronal death in the early phase of neural tissue damage [77]. After 48 h from of axonal damage, GPR17 receptors are exposed on the surface of activated microglial cells (brain-settled macrophages) located at the limit of the nerve tissue damage. These observations suggest that, initially, damage to the brain structures leads to the emergence of GPR17 on damaged neurons and then on activated immune cells. After 72 h of damage, there is a proliferation of the lining cells on which the presence of GPR17 is found, which emphasizes the activation of their de-differentiating towards the progenitor pluripotent cells. At the same time, OPCs proliferate in both the brain and spinal cord, and the process of rebuilding the myelin sheath (remyelination) begins [80]. One week after injury, the GPR17 level again increases in the area of damage, and is again associated with infiltration of macrophage cells that previously were found on the edge of the damage site. This effect lasts up to two weeks [82].

**Figure 1:**
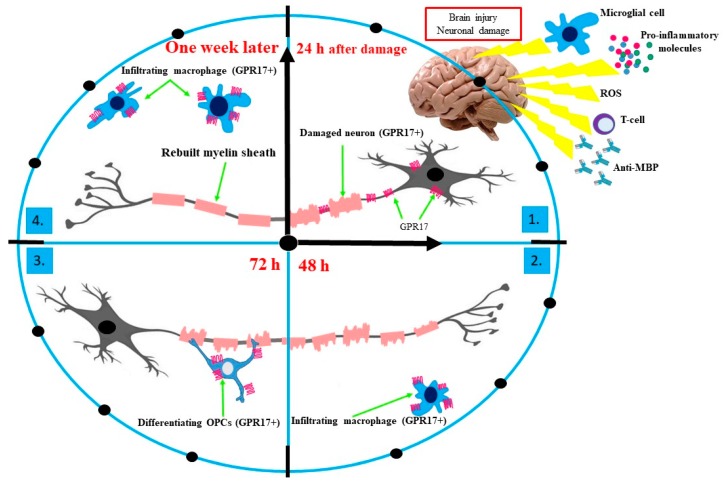
The role of GPR17 receptors as a specific marker of CNS damage. In the early stage of CNS tissue injury (within 24 h), an increased level of GPR17 is observed in damaged neurons and there is considerable growth in the mortality of GPR17+ neurons (**1**). Once 48 h have passed since axonal damage, GPR17 receptors are not located on injured neurons, but only at the limit of the nerve tissue damage due to exposition on the surface of infiltrating macrophages (activated microglial cells) (**2**) Once 72 h have passed since damage, a proliferation of OPCs (GPR17+) is observed. Expression of GPR17 on the surface of OPCs blocks their differentiation and maturation into myelinating OLGs. Myelination is possible after losing the GPR17 receptors by OLGs (**3**). One week after brain injury, the GPR17 level again enhances in the area of damage and is associated with the resumed massive infiltration of macrophage cells that previously resided on the edge of the damage site (**4**). Abbreviations: anti-MBP, anti-major basic protein; GPR17, G-protein coupled receptor 17; OPCs, oligodendrocyte precursor cells; OLGs, oligodendrocytes; ROS, reactive oxygen species.

The role of GPR17 receptors as a specific marker of CNS damage has been confirmed in the latest studies, in which the level of this receptor was determined in autopsy material or from neurosurgical samples collected from patients with brain injuries. These studies have demonstrated the presence of GPR17 receptors at and around the lesions in dying neurons, activated astrocytes, and macrophages of microglia. The level of GPR17 receptors has been positively correlated with the distance from the site of damage and the degree of damage, and negatively correlated with the time elapsed from the injury [92]. To summarize, in the early stages of oligodendrocyte differentiation, the presence of GPR17 receptors stops the cells in their immature form, which is necessary for the remyelination process. Then, after reaching the critical point, the differentiating cells inhibit the synthesis of GPR17, as the continuous activity of these receptors would inhibit cell maturation. The specific role of GPR17 in neuronal damage is shown in Figure 1.

**Figure 2:**
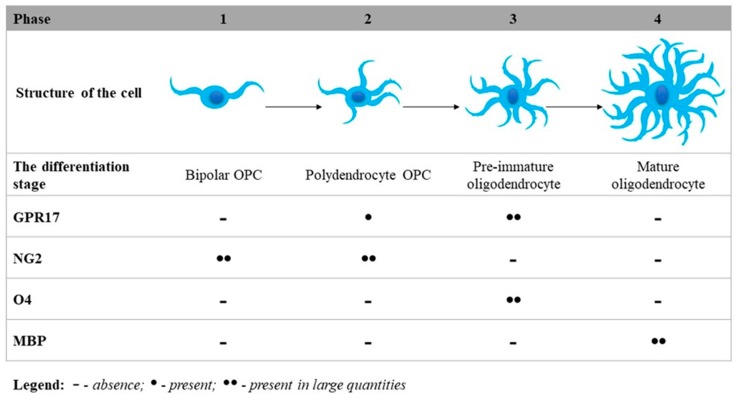
Phases of GPR17 differentiation. GPR17 as an inner timer of oligodendrocyte differentiation and the myelination process. GPR17 is expressed in the oligodendrocyte lineage cells, and its level is increased during differentiation of NG2+ OPCs into O4-pre-myelinating oligodendrocytes. It is evidently downregulated during the terminal maturation of myelinating oligodendrocytes, which express MBP.

Research conducted by Fumagalli et al. demonstrated several processes in a detailed way for the first time, including the typical differentiation steps of OPCs from immature precursors to mature oligodendrocytes; changes in the protein expression of typical pre-oligodendroglial markers such as GPR17, NG2, and O4 (O-antigens are expressed on oligodendrocytes during differentiation and on mature oligodendrocytes in the CNS); and markers of mature myelinating oligodendrocytes, such as myelin basic protein (MBP), which maintains the proper structure of myelin by interacting with the lipids in the myelin membrane. The results obtained by Fumagalli et al. clearly indicate that GPR17 can be considered a novel lineage marker indicating NG2+ cells (polydendrocytes) and pre-immature oligodendrocytes. At the primary stage, bipolar OPCs have already expressed mRNA transcripts for GPR17, but receptor protein expression is detectable only when NG2+ cells acquire a more complex morphology (known as polydendrocyte OPCs). Subsequently, GPR17 expression gradually increases in pre-immature oligodendrocytes, finally reaching a maximum level after displaying O4 antigen (pre-immature oligodendrocytes). In mature oligodendrocytes, GPR17 expression is turned down, and mature MBP+ cells no longer express the GPR17 receptors (mature oligodendrocytes) [106]. The differentiation steps of OPCs forming an immature precursor to the mature oligodendrocyte with an expression level of typical differentiation markers are illustrated in Figure 2.

There are studies that confirm that GPR17 receptors are important regulators of oligodendrocyte development and maturation [106,107,108,109]. During the early stage, OPC cells show negligible GPR17 receptor expression, which gradually increases as the cells mature. The GPR17 expression reaches a plateau in the pre-immature phase of oligodendrocytes then decreases during the final differentiation of oligodendrocytes. Accordingly, GPR17 co-expresses along with early NG oligodendrocyte markers, and this expression is down-regulated in cells with MBP expression [84,108]. MBP is the second-most abundant myelin protein after the proteolipid protein constituting the myelin sheath, which is indispensable in the normal activity of the CNS [110].

Chen et al. analyzed GPR17 overexpression in transgenic and knockout mice. The results they obtained demonstrated that GPR17 negatively regulates oligodendrocyte differentiation and myelination in vitro and in vivo in mice with experimental autoimmune encephalomyelitis (EAE), and in the spinal lesions of MS patients. The study by Chang et al. suggests that GPR17 functions as a powerful negative regulator for oligodendrocyte myelination by inducing nuclear localization of differentiation inhibitors ID2/4 [111]. However, a detailed study of GPR17 dysfunction under in vivo conditions associated with demyelination and inflammation has still not been conducted, nor is it known whether changes in the pool of GPR17+ cells contributes to or contrasts with lesion repair.

The basic helix-loop-helix (bHLH) transcription factors Olig1 as well as Olig2 play a significant role in oligodendrocyte functioning [112]. Olig2 takes part in the differentiation of oligodendrocyte lineage, whereas Olig1 is involved in the terminal stage of myelination. When Olig2 manages multipotent neural stem/progenitors to become lineage-restricted OPCs, Olig1 promotes final maturation of oligodendrocytes [113]. Furthermore, demyelination disrupts saltatory nerve conduction, leading to axonal damage and in consequence of irreversible neurological disabilities [114].

Extra- and intracellular signals such as cell cycle inhibitors (p21, p27, and p57) and oligodendrocyte differentiation inhibitors (ID2 and ID4) affect the regulate maturation of oligodendrocyte cyclin-dependent kinase inhibitors (CKIs) through proteins regulating various steps of neurogenesis. Two groups of CKIs promote cell cycle disruption through inhibiting the activity of cyclin/cyclin-dependent kinase (CDK) complexes: the Cip/Kip family (p21Cip1, p27Kip1), and p57Kip2. Cip/Kip proteins are also involved in the differentiation of glial cells, including oligodendrocytes [115,116]. Furthermore, under physiological conditions, oligodendrocyte lineage cells upregulate ID2 and ID4, which results in blocking the expression of immature/mature oligodendrocyte markers in vitro. This indicates that ID2 and ID4 may be associated with blocking oligodendroglial development [117,118].

GPR17 receptors play a diverse role depending on the time and place of their occurrence. Nucleotide receptors can act as ‘sensors’ activated after a brain injury, participating in both the death of neurons as well as the repair response of oligodendrocytes. In addition, GPR17 receptors are important markers of the individual developmental stages of oligodendrocytes.

## 4. Conclusions

The development of neurodegenerative diseases, including MS, is a complex phenomenon, the mechanisms of which are not yet fully understood. It is extremely important to constantly search for information on the factors responsible for the formation, course, and treatment of these diseases. MS is an immune-mediated inflammatory disease that is difficult to monitor due to the heterogeneity of its clinical symptoms, its rate of progression, and its response to therapy, which reflects the existence of several pathogenic mechanisms. The initially diagnosed RR stage is characterized by periods of exacerbation (with acute inflammation) and remission. In the development of the disease, RRMS evolves into the SP stage, characterized by the progression of neurodegeneration. Both chronic and recurrent microglia inflammation facilitate demyelination and axon degeneration. CNS microglia is considered a typical environment for antigen-presenting cells that can up- and downregulate the expression of the immune response. However, the molecular processes leading to axonal and neuronal injury in MS are not understood. GPR17 nucleotide receptors could be an important element in our understanding of the molecular mechanisms underlying the demyelinating processes in MS. GPR17 can play a dual role, differing in the time and place of its occurrence in various types of cells. According to the latest reports, GPR17 receptors play a role in both the demyelination and remyelination occurring in CNS. Receptors can contribute to the death of neurons present inside the inflammatory focus, as well as causing local nerve tissue repair. GPR17 receptors are also an ideal marker of oligodendrocyte development, while uracil and CysLT nucleotides have been recognized as the main factors regulating the development of OPCs. These receptors could be the focal point for future therapies designed to accelerate the regeneration of nerve cells and thwart the demyelination process.

## Figures and Tables

**Table 1:** Types of Gα-protein and potential effects on the human body.

G α-Subunits	Signal Transduction	Receptors	Effects on Human the Body	Ref.
Gα_i_	inhibition of AC; activation of phosphodiesterase; open K^+^ channels; closes Ca^2+^ channels	α2-adrenergic receptors; chemokine receptors; serotonin receptors; histamine receptors; dopamine receptors; rhodopsin; taste receptors; platelet’s receptors	smooth muscle contraction; depression of neuronal activity; vision; taste; maintains ionic balance; cell motility	[50,51,52,53]
Gα_s_	activation of AC	βa-adrenergic receptors; serotonin; dopamine receptors; histamine receptors; olfactory receptors	smooth muscle relaxation; stimulation of neuronal activity; smell; cell growth and motility; Ca^2+^ influx	[51,53,54,55]
Gα_q/11_	inhibition of AC	α1-adrenergic receptors, endothelin receptors; vasopressin receptors; chemokine receptors	intracellular calcium mobilization; smooth muscle cell proliferation	[56,57,58,59]
Gα_12/13_	activation of the Rho family of GTPases	-	platelet activation; neuronal activity; immune response (e.g., lymphocyte adhesion and migration, neutrophil chemokinesis and chemotaxis); cell polarity; cell shape; changes in the cytoskeleton	[60,61,62]
